# Regenerative Therapy in Osteoarthritis Using Umbilical Cord-Origin Mesenchymal Stem Cells: A Critical Appraisal of Clinical Safety and Efficacy Through Systematic Review and Meta-Analysis

**DOI:** 10.1155/sci/4261166

**Published:** 2025-12-02

**Authors:** Elnaz Lohrasbi, Soraya Babaie, Hadi Hamedfar, Samira Pourzeinali, Azizeh Farshbaf-Khalili, Vahideh Toopchizadeh

**Affiliations:** ^1^Physical Medicine and Rehabilitation Research Center, Aging Research Institute, Tabriz University of Medical Sciences, Tabriz, Iran; ^2^Amiralmomenin Hospital of Charoimagh, Vice Chancellor for Treatment, Tabriz University of Medical Sciences, Tabriz, Iran

**Keywords:** efficacy, mesenchymal stem cells, meta-analysis, osteoarthritis, regenerative therapy, safety, umbilical cord

## Abstract

**Introduction:**

Recent decades have witnessed a high prevalence of knee osteoarthritis among adults, which is associated with chronic pain, functional limitations, and decreased quality of life. Given the ineffectiveness of conventional cartilage regeneration approaches, umbilical cord-derived mesenchymal stem cells (UC-MSCs) have emerged as a potential regenerative therapy. In this study, it was aimed to determine whether UC-MSC treatment for knee osteoarthritis is effective, safe, and what is the optimal dosage to achieve optimal outcomes.

**Methods:**

This study was conducted as a systematic review and meta-analysis based on the PRISMA 2020 guideline. The dose of cells was divided into four groups: less than 25 × 10^6^ (low), 25–50 × 10^6^ (medium), more than 50 × 10^6^ (high), and cases with no dose reported. An extensive search was conducted in PubMed, Embase, Scopus, Web of Science, ClinicalTrials.gov, and other sources up to September 2025. Randomized, phase I/II, and quasi-experimental clinical trial studies that used UC-MSCs in human patients with osteoarthritis were included in the analysis. Cochrane RoB and ROBINS-I tools were used to assess the quality of studies, and statistical analysis was performed using RevMan 5.4.

**Results:**

Of the 1427 identified articles, 8 studies with a total of 688 participants were finally included in the systematic review and meta-analysis. Although the analysis indicated that intra-articular injections of UC-MSCs reduced pain intensity (visual analog scale [VAS]) at both 6 months (standardized mean difference [SMD]: −0.86; 95% CI: −2.41 to 0.69; *p*=0.28) and 12 months (SMD: −1.02; 95% CI: −2.62 to 0.58; *p*=0.21), the observed reductions were not statistically significant. Notably, a subgroup analysis revealed that administration of a low-dose UC-MSC formulation resulted in a statistically significant reduction in pain scores at both 6 and 12 months (*p* ≤ 0.0001). In addition, there was also an improvement in the Western Ontario and McMaster Universities Osteoarthritis Index (WOMAC) total score at 6 months (SMD: −25.81; 95% CI: −45.15 to −6.46; *p*=0.009) compared to control. Compared to high-dose groups or control interventions (e.g., hyaluronic acid or placebo), low and medium doses (less than 25 × 10^6^ and 25–50 × 10^6^ million cells) demonstrated a superior efficacy. No serious treatment-related adverse events were reported, and the adverse events were mild, transient, and manageable.

**Conclusion:**

UC-MSC treatment in patients with knee osteoarthritis is a safe and effective method for improving pain and motor function, and it represents a promising alternative to traditional treatments. Given the promising results of this study, further prospective studies with a standardized design and economic evaluations are recommended to enable wider clinical application of this method.

## 1. Introduction

Osteoarthritis is a chronic, debilitating joint disease with a highly increasing rate of incidence that primarily affects middle-aged and older adults and has substantial social and economic implications [1]. As the disease progresses, articular cartilage is gradually destroyed, chronic inflammation develops, the subchondral structure changes, and eventually joint mobility is impaired [2]. The increasing prevalence of this disease worldwide, especially with the increase in the average age of the population, has led the World Health Organization to introduce it as one of the main causes of physical disability in adults [3]. In addition, osteoarthritis leads to a decrease in quality of life, functional dependence, depression, and imposes heavy costs on health systems and households [4]. The importance of this disease is doubled because, so far, no definitive treatment has been identified to stop or reverse its progression, and existing treatments are mainly limited to relieving symptoms and reducing pain [5]. Recently, stem cell-based therapies have been considered as a new approach for cartilage regeneration based on regenerative medicine and tissue engineering. It has been found that mesenchymal stem cells (MSCs) are particularly useful in preclinical and clinical studies, due to their ability to differentiate into different cell lineages, including chondrocytes, and their immunomodulatory qualities [6, 7]. One of the most important sources of MSCs is fetal tissues or fetal derivatives such as yolk sac, umbilical cord, Wharton's jelly, and amniotic fluid, which have potential advantages over adult MSCs, such as greater proliferation capacity, higher biological potential, and reduced risk of cellular senescence [8]. These characteristics have led to the emergence of umbilical cord-derived MSCs (UC-MSCs) as a promising option, especially in degenerative diseases such as osteoarthritis. It is still unclear whether these types of cells are safe and effective in treating osteoarthritis, despite promising data from early studies [9]. The cells have been shown to reduce pain, improve joint function, and promote cartilage regeneration in some studies, while others have shown limited efficacy or concerns about immune reactions, off-target differentiation, or even the development of neoplasms [10]. These conflicting study results, combined with the heterogeneity of designs, target populations, and evaluation indicators, have made it difficult and challenging to make a definitive judgment about the clinical utility of this treatment [11]. It is necessary to systematically synthesize existing data through systematic reviews and meta-analyses in order to be able to provide a clear, transparent, and evidence-based picture of the effectiveness and safety of this treatment. A systematic review and quantitative analysis of empirical data on UC-MSCs in the treatment of osteoarthritis is conducted in the present study to fill this scientific gap and resolve existing ambiguities. The strength of this study is that it purposefully and with an analytical approach, simultaneously examined the efficacy and safety aspects of these cells based on dose response and also attempted to identify and control sources of heterogeneity and possible bias using advanced statistical methods.

## 2. Methods and Materials

### 2.1. Study Design

This systematic review and meta-analysis were designed and conducted according to the PRISMA 2020 (Preferred Reporting Items for Systematic Reviews and Meta-Analyses) guidelines. The study protocol was prewritten and registered in the PROSPERO database (registration ID: CRD420251112952).

### 2.2. Study Search Strategy

A comprehensive and systematic search was conducted in electronic databases, including PubMed (MEDLINE), Embase, Scopus, Web of Science, ScienceDirect, ProQuest, ClinicalTrials.gov, and Cochrane CENTRAL, from the beginning of each database until September 2025. The search strategy included a combination of MeSH terms and free keywords in English, including the following: (“Mesenchymal stem cells” OR “umbilical cord” OR “cord, umbilical” OR “uniculus umbilicalis”) AND (“osteoarthritis” OR “degenerative arthritis” OR “degenerative joint disease” OR “noninflammatory arthritis” OR “arthritis, degenerative” OR “arthritis, noninflammatory” OR “arthrosis” OR “osteo-arthritis” OR “osteo-arthrosis” OR “osteoarthrosis” OR “primary osteoarthritis” OR “rheumatoid arthrosis”) AND (“clinical trial” OR “clinical drug trial” OR “major clinical trial” OR “trial, clinical” OR “controlled clinical trial” OR “clinical trial, controlled” OR “controlled clinical comparison” OR “controlled clinical drug trial” OR “controlled clinical experiment” OR “controlled clinical study” OR “controlled clinical test” OR “controlled clinical trial” OR “RCT” OR “randomized controlled trial” OR “controlled trial, randomized” OR “randomized controlled study” OR “randomized controlled trial” OR “randomized controlled study” OR “trial, randomized controlled”).

To increase precision, filters were applied to limit results to human studies and clinical studies, including both randomized and nonrandomized trials. No restrictions were applied in terms of language, publication status, or year of study. This study was approved by the Ethical Committee of Tabriz University of Medical Sciences (Ethics Number: IR.TBZMED.VCR.REC.1403.135).  Table 1 shows the PICO strategy used to establish the guiding question of this review.

### 2.3. Inclusion and Exclusion Criteria

All study designs, such as RCTs, phase I/II trial studies, quasi-experimental studies, and single-arm interventional studies conducted on human subjects with osteoarthritis with a clinical or imaging diagnosis and used human UC-MSCs for therapeutic intervention, regardless of the route of administration (intra-articular, intravenous, etc.), were included. Additionally, reporting at least one efficacy (e.g., pain reduction, improvement in joint function, imaging changes) or safety (adverse events, immune response, etc.) was also another important criterial for study inclusion. Nonhuman studies (animal or cell-based), literature reviews, conference abstracts without full text, studies that used other sources of MSCs (e.g., bone marrow, adipose tissue), and studies that did not provide detailed information on cell origin, administration method, or extractable outcomes were all excluded from the systematic review.

### 2.4. Study Selection and Data Extraction

A predesigned extraction form was used by two authors to independently screen the literature and extract the data. The following data were extracted: the first author's name, journal, year of publication, country of study, study design, sample size, participants (e.g., age, sex, disease severity, joint location involved), intervention details (e.g., exact source of MSC, injection dose, number of injections, administration method, follow-up), comparator, outcomes assessed (visual analog scale [VAS], Western Ontario and McMaster Universities Osteoarthritis Index [WOMAC], Knee Injury and Osteoarthritis Outcome Score [KOOS], MRI findings, etc.), adverse events and safety (reported side effects, severe side effects, inflammatory or immune responses). Disagreements between the two reviewers were resolved by discussion and, if necessary, by a third researcher's judgment (Table 2).

### 2.5. Assessment of Study Quality

To assess the quality of the included studies, the Cochrane Risk of Bias tool 2.0 was used for RCT studies and the ROBINS-I tool for nonrandomized studies. The two reviewers independently performed these assessments, and disagreements were resolved by consultation.

### 2.6. Meta-Analysis

Statistical analysis was performed using RevMan version 5.4.1 (version 5.4; The Nordic Cochrane Centre, Copenhagen, Denmark). For continuous data, the standardized mean difference (SMD) was used. Heterogeneity between studies was measured using the I^2^ statistic. In case of high heterogeneity (*I*^2^ > 50%), the random-effects model was used. The dose of cells was divided into four groups based on the results of a recent study [18]: less than 25 × 10^6^ (low), 25–50 × 10^6^ (medium), more than 50 × 10^6^ (high), and cases with no dose reported. Studies were sub-grouped based on the received dose.

## 3. Results

### 3.1. Study Selection

**Figure 1:**
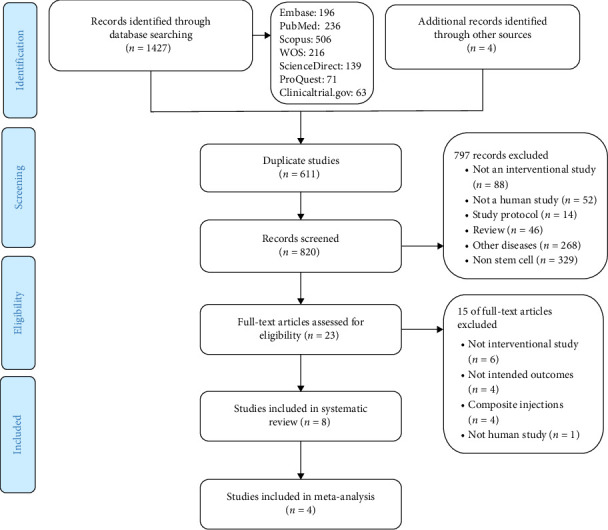
Flow diagram of the study process.

As a result of the initial search, 1427 articles were identified, out of which 820 studies were considered for title and abstract review after 611 duplicates were removed. The full-text of 28 articles was evaluated after 792 articles were excluded due to noncompliance with the inclusion criteria. Ultimately, 8 studies were eligible for systematic review [12–19], and 4 studies for meta-analysis [13–15, 17] (Figure 1).

### 3.2. Characteristics of Included Studies

A total of 688 participants were included in the eight studies published between 2016 and 2024 included in the systematic review. Different countries conducted the studies, including Australia, Chile, China, USA, Iran, and Jordan, indicating an international distribution and multidisciplinary nature of the studies. Many studies were double-blind RCTs [12–15, 17], while others were prospective phase I/II studies [19], dose-response trials [18], or single-arm studies [16]. A total of 508 patients received MSCs (Intervention group), while 180 received either hyaluronic acid, corticosteroid injections, or placebos (saline normal) (control groups). In the studies, sample sizes ranged from 14 to 480, with male and female participants ranging in age from 40 to 75. According to Kellgren–Lawrence classification, the majority of patients had knee osteoarthritis of grades II–IV. MSCs were injected intra-articularly in all studies. Human umbilical cord tissues, Wharton's gel, and cord blood were the main sources of cells, although placental cells were also used in one study. There was also a variety of methods used for cell preparation across studies, including frozen or fresh cells, with ex vivo expansion in vitro. Most studies used conventional doses of 20 or 40 million cells, but some used doses ranging from 2 to 80 million cells. Dose-response design was employed in one study, comparing low, medium, and high doses (2, 20, and 80 million cells) [18]. Many studies assessed patients' pain and function as primary outcome with standard instruments such as VASs, WOMAC indexes, KOOS and Lysholm scores, and joint ranges of motion (ROM). Secondary outcomes included structural assessment by MRI (using scores such as magnetic resonance observation of cartilage repair tissue [MOCART] and whole-organ magnetic resonance imaging score [WORMS]), and quality of life by instruments such as SF-12, SF-36, and EQ-5D. The follow-up duration in the studies ranged from 3 to 48 months, and most reported data for 6–12 months. MSC was not associated with any serious adverse events following inclusion in any of the studies. Joint swelling, pain at the injection site, or effusion were the most commonly reported adverse events. These were typically mild and transient, and they usually resolved by themselves or with supportive care (Table 2).

### 3.3. Risk of Bias Assessment

**Figure 2:**
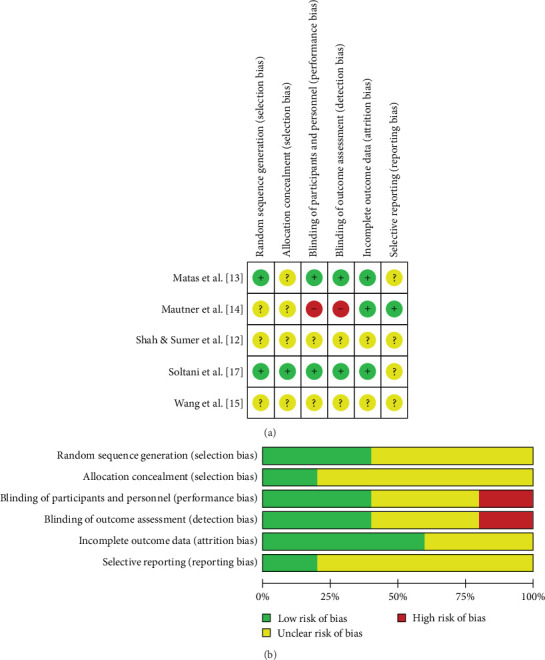
(A) Risk of bias summary: review authors' judgments about each risk of bias item for each included study (for umbilical cord randomized studies). (B) Risk of bias graph: review authors' judgements about each risk of bias item presented as percentages across all included studies.

Risk of bias assessment for randomized studies was performed using the Cochrane RoB tool. Most studies were at low to moderate risk of bias in terms of randomization, blinding, and outcome assessment (Figure 2a,b). For quasi-experimental studies, assessment using the ROBINS-I tool showed that three studies had an overall moderate risk of bias; this was mainly due to bias due to confounding variables and deviations from the planned intervention (Table 3).

### 3.4. Meta-Analysis Results

#### 3.4.1. Comparison of Pain Scores (VAS) at 6 Months

**Figure 3:**
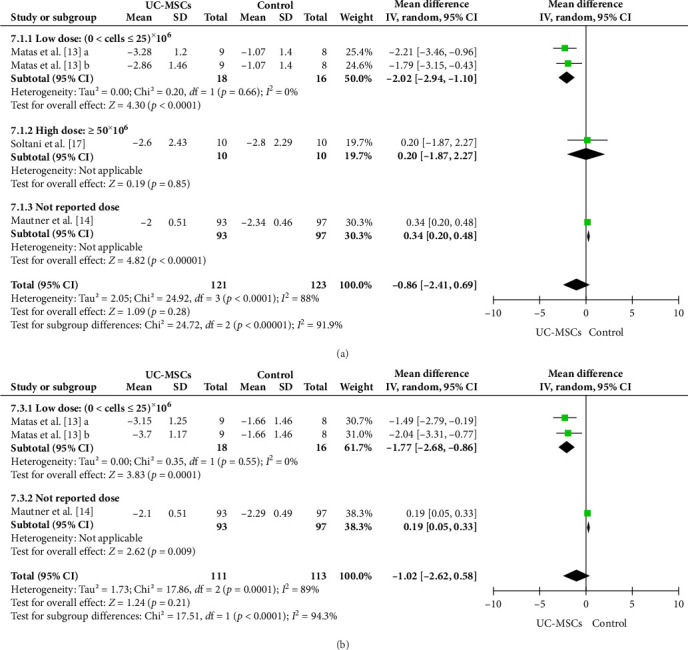
(A) Forest plot of VAS score comparing UC-MSCs and control at 6 months. (B) Forest plot of VAS score comparing UC-MSCs and control at 12 months. In the Matas et al. [13] study with three arms, in one intervention arm (a), one injection of UC-MSCs was performed at the beginning of the study. In the second intervention arm (b), in addition to the baseline, a second injection was performed at 6 months.

Based on data from the included studies (consisted of three studies and four outcomes), the meta-analysis showed that intra-articular injection of UC-MSCs reduced pain intensity after 6 months compared with the control group. The combined effect size (SMD) was −0.86 with a 95% confidence interval of −2.41 to 0.69. This difference was not statistically significant (*p*=0.28). However, using low-dose UC-MSCs showed a significanct reduction in pain score. The heterogeneity of the studies was also moderate to high (*I*^2^ = 88%), which could be due to differences in cell dose, study design, and patient population (Figure 3a).

#### 3.4.2. Comparison of Pain Scores (VAS) at 12 Months

In the analysis performed for the VAS score at the end of 12 months, the results indicated the continued effectiveness of UC-MSCs treatment in reducing pain. The combined effect size at this time was −1.02 with a 95% confidence interval of −2.62 to 0.58, which was not a statistically significant difference (*p*=0.21). However, using low-dose UC-MSCs showed significanct reduction in pain score (Figure 3b). Heterogeneity between studies at this time was also relatively high (*I*^2^ = 94.3%), which is likely due to differences in injection doses and frequency of intervention.

#### 3.4.3. WOMAC Total Score Comparison at 6 Months

**Figure 4:**
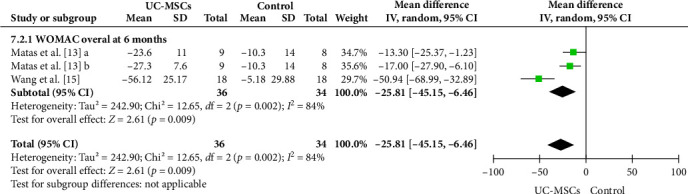
Forest plot of WOMAC overall score comparing UC-MSCs and control at 6 months. In the Matas et al. [13] study with three arms, in one intervention arm (a), one injection of UC-MSCs was performed at the beginning of the study. In the second intervention arm (b), in addition to the baseline, a second injection was performed at 6 months.

In assessing functional outcomes via the WOMAC composite index at 6 months, the meta-analysis data showed a significant reduction in the WOMAC total score in the UC-MSCs group compared with the control. The combined effect size was −25.81 with a 95% confidence interval of −45.15 to −6.46 (*p*=0.009). The amount of heterogeneity in this analysis was also high (*I*^2^ = 84%), which may be related to differences in data collection criteria, variation in treatment protocols, and time points (Figure 4).

Overall, the results of the meta-analysis suggest the effectiveness of UC-MSCs treatment in reducing pain and improving function in patients with knee osteoarthritis in the short (6 months) and medium (12 months) terms. However, the high level of heterogeneity between studies highlights the need for prospective studies with more standardized designs and more homogeneous populations. None of the included studies reported serious adverse events related to the intervention. Mild adverse events such as local pain or swelling after injection, transient effusion, or joint discomfort were reported in some studies, which often resolved with rest or symptomatic treatment.

### 3.5. Quality of Life

Several studies have investigated the quality of life of patients with knee osteoarthritis after injection of UC-MSCs. In the randomized, phase I/II trial by Matas et al. [13], quality of life was assessed using the SF-36 questionnaire. Results showed that patients receiving two MSC injections (MSC-2) had greater improvements in pain and function-related indices at 12-month follow-up compared with the control group (hyaluronic acid), although no significant changes were seen on MRI imaging [13]. In the large trial by Mautner et al. [14] with 480 patients, quality of life was assessed using the EQ-5D and PROMIS-29 instruments. Analyses showed that there were no significant differences in quality of life over time between the treatment groups (UC-MSCs, autologous bone marrow aspirate concentrate [BMAC], SVF, and corticosteroids), and similar patterns of change were observed among all groups [14]. The study by Wang et al. [15] also assessed quality of life using the SF-36 scale. The results indicated that in the MSC group, SF-36 scores improved significantly from the second to the sixth month after treatment, and at the third and sixth months, this improvement was significantly better than in the control group (hyaluronic acid). In contrast, the control group did not show a significant change in quality of life [15]. Finally, the study by Ao et al. ([16], China), which was a single-arm phase I study, assessed quality of life using the SF-12 questionnaire. At 3-month follow-up after four injections, the SF-12 score significantly increased (from 39.0 [35.8–42.3] to 46.0 [44.0–48.3]), indicating a significant improvement in patients' quality of life [16].

### 3.6. Results of Studies Included in the Systematic Review (Nonmeta-Analysis)

Four prospective studies that were not included in the meta-analysis examined the effects of cord blood-derived MSC injections in patients with knee osteoarthritis without the control group [12, 16, 18, 19], but their qualitative review provides valuable information on the safety and efficacy of cord blood-derived MSCs in osteoarthritis. In a study by Shah and Sumer [12] in Australia, 29 patients underwent MSC injections in a randomized, double-blind, placebo-controlled trial. Patients receiving two injections (40 million cells each: medium dose) showed significant improvements in pain reduction (86% vs. 38%) and disability reduction (89% vs. 50%) over 12 months of follow-up compared to the control group receiving hyaluronic acid, while side effects included only mild cases of transient synovitis [12]. Ao et al. [16] conducted a single-arm phase I study in China, and 14 patients were treated with 4 weekly injections of umbilical cord MSC (1.5 × 10^7^ cells each time). At 3-month follow-up, WOMAC and VAS scores significantly decreased, and quality of life (SF-12) and MRI (MOCART) indices improved. Adverse events occurred in 35.7% of patients, mainly joint pain and swelling, and were all reported to be transient and nonserious [16]. In the study by Matas et al. (2024) in Chile, 40 patients were assigned to three low-dose (2 × 10^6^ cells), medium-dose (20 × 10^6^ cells), and high-dose (80 × 10^6^ cells) umbilical cord MSC groups. All groups had significant improvements in WOMAC and VAS at 3 and 6 months, although the efficacy was greater in the low-dose and medium-dose groups than in the high-dose group. WORMS assessment did not show any significant changes in cartilage structure. In terms of safety, all doses were tolerated, but in the high-dose group all patients experienced transient joint pain and swelling [18]. Finally, Samara et al. [19] in Jordan, in a prospective phase I/II study, treated 16 patients with moderate to severe osteoarthritis with two injections of MSC derived from Wharton's jelly (mean 42–44 million cells per injection). In long-term follow-up up to 48 months, significant improvements in functional indices (KOOS) and pain reduction were observed. In addition, MRI at 12 months showed a significant reduction in cartilage lesions, osteophytes, bone marrow lesions, and inflammatory signs. Only two nonserious complications were reported, including moderate effusion and superficial phlebitis [19]. These four studies demonstrate that the injection of UC-MSCs or placenta-derived MSCs is safe and largely effective in improving pain, function, and quality of life in patients with knee osteoarthritis.

## 4. Discussion

As a result of the systematic review and meta-analysis, UC-MSCs are not only effective in reducing pain and improving motor function when used as a regenerative therapeutic approach for knee osteoarthritis, but they are also safe and tolerable, with mild and transient adverse events often resulting from their use. The analysis revealed that while pain reduction (VAS) at 6 and 12 months was not statistically significant compared to the control, a marked decrease occurred after low-dose injection. Furthermore, enhancements in the joint function score (WOMAC) at 6 months demonstrated superior outcomes in the UC-MSC group compared to controls. In addition to the studies included in the meta-analysis, four other studies were identified that were not included in the final analysis due to a lack of a control group or heterogeneity in outcome assessment. The results of these studies generally demonstrated significant improvements in patients' quality of life after receiving stem cell-based interventions, with reported indicators including reduced pain, improved functional capacity, and enhanced motor abilities [12, 16, 18, 19]. Beyond these clinical benefits, however, MRI outcomes for UC-MSC therapy in knee osteoarthritis remain inconsistent across studies. While two studies [16, 19] reported structural improvements in cartilage and subchondral integrity, another [18] showed no significant change. Although the lack of a comparison group prevented a precise assessment of the extent of the intervention's effect, the alignment of the findings with the results of the studies included in the meta-analysis provides further evidence in support of the effectiveness of this therapeutic approach. However, the lack of appropriate controls and the variability in quality-of-life measurement tools limit definitive conclusions from these data.

UC-MSCs play a reparative and palliative role in knee osteoarthritis through several diverse mechanisms [20]. One of the most important pathways is immune regulation and reduction of local inflammation [21]. UC-MSCs are able to reduce the chronic inflammatory response of the joint by secreting anti-inflammatory cytokines such as IL-10 and TGF-*β* and inhibiting proinflammatory cytokines such as TNF-*α* and IL-1*β*, which leads to a reduction in pain and swelling [9]. In addition, UC-MSCs provide a suitable environment for cartilage regeneration by secreting paracrine growth factors such as VEGF, IGF-1, and FGF, increasing the survival of native chondrocytes and stimulating the production of extracellular matrix [20]. These factors also play a role in improving local angiogenesis and nutrition of joint cells, and preventing further cartilage destruction [22]. Beyond their direct effects, UC-MSCs act through a paracrine mechanism to enhance the activity of native joint cells and fibroblasts. This process facilitates partial cartilage repair, reduces osteophytes, and promotes subchondral bone remodeling [23, 24]. Some animal and preclinical studies have also shown that these cells can slow the progression of osteoarthritis by inhibiting chondrocyte apoptosis and reducing cartilage matrix degradation [24–26].

In order to investigate the effects of MSCs on pain and joint function improvement in patients with knee osteoarthritis, several studies have compared different sources of MSCs. Recent meta-analyses have shown that adipose-derived MSCs (AD-MSCs) are effective in reducing pain (using VAS scale) and improving joint function (using WOMAC scale), and in some reports their clinical effects are superior to those of bone marrow–derived mesenchymal stromal cells (BM-MSCs) or UC-MSCs [27–30]. For example, one study showed that intra-articular injection of AD-MSCs significantly reduced patients' pain and improved motor function, while no significant difference was observed between AD-MSCs and BM-MSCs in improving function [29]. Also, other studies showed that different doses of MSC (such as 2 × 10^6^, 20 × 10^6^, and 80 × 10^6^ cells) had similar therapeutic effects on pain and joint function, but intermediate doses were identified as the optimal dose with a good balance between efficacy and local side effects [31]. These findings are consistent with the results of the studies included in our meta-analysis (which mainly used UC-MSCs) and suggest that the selection of the appropriate cell source and dose can help improve the safety and efficacy of the treatment. The evaluation of structural outcomes via MRI reveals a complex and varied picture of UC-MSC therapy's impact on knee osteoarthritis. While Samara et al. [19] reported significant improvement across multiple structural domains including cartilage integrity, osteophyte reduction, and resolution of bone marrow lesions at 12 months [18], other studies such as Matas et al. [13] found no significant structural changes using the WORMS scoring system [19], and Ao et al. [16] observed improved cartilage repair tissue morphology per the MOCART score, albeit at a short 3-month follow-up. These discrepancies may be attributed to differences in MRI assessment tools, with repair-oriented systems like MOCART potentially capturing subtle regenerative changes more effectively than degeneration-focused tools like WORMS [32, 33], as well as variations in follow-up duration, cell dosage, and injection protocols. Longer observation periods and standardized imaging methodologies are essential to more reliably determine the structural regenerative potential of UC-MSC therapy.

One of the most important issues examined in this study was determining the optimal dose of stem cells to achieve the greatest therapeutic effect with the least side effects. A review of the included studies showed that although higher doses (e.g., 80 million cells) were sometimes associated with a faster therapeutic response, they were also associated with an increased likelihood of local complications such as swelling and pain at the injection site [12, 18, 19]. On the other hand, medium doses were reported in most studies with promising efficacy and fewer side effects, which could be a basis for determining an effective and safe dose in the future [12, 18, 19]. Although one of the aims of this systematic review was to investigate the optimal dose of UC-MSCs for the treatment of knee osteoarthritis, the included studies provided limited and heterogeneous data in this area. Only a few trials directly compared different doses, and these studies varied greatly in terms of the number of cells injected, the frequency of injections, and the duration of follow-up. Therefore, the available evidence is insufficient to draw definitive conclusions about the optimal dose, and well-designed clinical trials with direct dose comparisons are needed.

From a safety perspective, none of the studies included in this systematic review reported severe or life-threatening adverse events. Common side effects included injection site pain, transient effusion, or mild joint stiffness, which often resolved with symptomatic treatment or without intervention [34]. These findings suggest an acceptable and stable safety profile of this type of treatment, especially compared with some pharmacological or surgical methods that are associated with systemic or long-term side effects [35]. Another issue of interest was patient satisfaction and quality of life after treatment [36]. Although many included studies did not directly measure patient satisfaction, improvements in SF-12, SF-36, and EQ-5D scores in some studies indicated improved general functioning, reduced functional limitations, and increased quality of life after receiving stem cells [13–16]. It should be noted that some patients have returned to levels of daily activity that were impossible or limited before treatment, with improved pain and function.

The cost-effectiveness of UC-MSCs can be assumed in the long term to be economically viable, even though detailed economic information was not provided in most of the included studies. As a result of reduced need for knee replacement, lower long-term anti-inflammatory and analgesic medication use, and improved motor function for patients, more invasive interventions like knee replacement are reduced [37]. This issue needs to be examined in more detail through prospective economic studies. Finally, it should be noted that this systematic review faced challenges, such as heterogeneity of studies, differing doses, study designs, and outcome assessment methods, despite the promising results. These issues can limit the interpretation of the results. Furthermore, insufficient long-term data and no standard guidelines exist for reporting adverse events, underlining the importance of high-quality clinical trials, longer follow-up periods, and the creation of standardized treatment protocols.

## 5. Conclusion

The present systematic review and meta-analysis provide evidence supporting the use of intra-articular UC-MSCs for improving function in knee osteoarthritis, with a trend toward pain reduction that did not reach statistical significance. In addition to clinical efficacy, this therapy has a favorable safety profile, mild adverse events, and relative patient satisfaction. Regarding the applied dose among included studies, the administration of a low-dose UC-MSC formulation resulted in a significant reduction in pain scores at both 6 and 12 months. The results also suggest that compared to high-dose groups or control interventions (e.g., hyaluronic acid or placebo), low and medium doses (less than 25 × 10^6^ and 25–50 million cells) demonstrated a superior efficacy. This review was able to paint a clearer and evidence-based picture of the place of UC-MSCs in the management of knee osteoarthritis. These results can provide effective guidance for physicians by highlighting a potentially optimal dosing strategy and confirming the treatment's strong safety and functional benefits.

## Figures and Tables

**Table 1:** PICOS criteria for inclusion and exclusion of studies.

Parameters determined the criteria for the present study	
Participants	People with osteoarthritis without restrictions in age and sex.
Intervention	The use of Umbilical vein mesenchymal stem cells without restrictions on dose or duration of intervention.
Comparator	Any control group, such as placebo, platelet-rich plasma (PRP) hyaluronic acid, corticosteroids, or no intervention.
Outcomes	Efficacy based on optimal dose and safety were as primary outcomes, as well as cost-effectiveness was secondary outcome.
Study design	Randomized controlled clinical trials, quasi-experimental studies, and single-arm interventional studies.

**Table 2:** The characteristics of the included studies investigating effects of umbilical cord mesenchymal stem cells on the knee osteoarthritis.

Study ID	Authors (year)	Country	Study design	Participants (e.g., number of participants, age, gender)	Intervention details (e.g., MSC dosage, duration of supplementation)	Comparator (if applicable)	Dose of MSCs	Source of MSCs	Outcomes assessed (both primary and secondary outcomes)	Results data (e.g., mean, standard deviation, confidence intervals)	Adverse events and safety
1: [12]	Shah K and Sumer H (2019)	Australia	Randomized, blinded, placebo-controlled trial.	29 patients with knee Osteoarthritis in three cohorts (1:1:1).	Intervention 1 received 1 injection of 40 million injections at 0 month and 3 mL of placebo (normal saline with 5% AB serum) at 6 months. Intervention 2 received 2 injections of 40 million at 0 and 6 months.	Control group received a conventional injection of hyaluronic acid.	40 million injection at 0 and 6 months.	Allogeneic mesenchymal stromal cells derived from umbilical cord.	Follow-up assessment for safety and efficacy at week 1, 4, 8, 12, 24, 36, and 52 weeks consisted of: WOMAC and VAS pain scoring system, the study reported at 12 months.	Patients in intervention 2 (group receiving two injections of MSCs) experienced 86% pain reduction and 89% disability reduction (*p*=0.001) contrasting to 38% and 50% in the control group.	No severe adverse events. Moderate adverse events in few patients comprised acute synovitis that resolved during 1 week following general analgesics and rest. Safety was confirmed due to no detection of alloantigen.

2: [13]	Matas J et al. (2018)	Chile	Randomized phase I/II trial.	Twenty-nine patients aged 40–65 years with Kellgren I–II–III knee-OA (three groups).	MSC1 group (*n* = 9) received UC-MSC injection only at baseline followed by placebo at 6 months, while MSC2 group (*n* = 9) received UC-MSCs at baseline and 6 months later.	Active control group (*n* = 8) received hyaluronic acid (HA) at baseline and 6 months later.	MSC injections contained 20 × 10^6^ UC-MSCs in 3 cc of saline with 5% AB plasma.	Umbilical cord-derived MSC (UC-MSC) 20 × 10^6^ cells (Cellistem-OA)	The primary endpoint was the safety of UC-MSC treatment. The secondary endpoint was VAS pain scale, WOMAC score, quality of life by the short-form 36 (SF-36) questionnaire, and MRI (WORMS score).	At 12 months, the MSC-2-treated group experienced significantly lower pain levels on the WOMAC-A (pain subscale) (1.1 ± 1.3) compared to the HA group (4.3 ± 3.5; *p*=0.04). The Pain VAS score was also significantly lower in the MSC-2 group than in the HA group (2.4 ± 2.1 vs., 22.1 ± 9.8, *p*=0.03) at 12 months. Additionally, the total WOMAC score was lower in the MSC-2 group than in the HA group at 12 months (4.2 ± 3.9 vs., 15.2 ± 11, *p*=0.05). No significant changes were displayed on MRI, measured by WORMS score.	Upon the initial injection, acute knee effusion was observed in 33% of cases in groups MSC-1 and MSC-2, compared to only 22% in the HA group (*p*=0.99). In second injection, 44% of patients in the MSC-2 group experienced knee effusion, compared to 37.5% in the HA group (*p*=0.99). Pain was the second most common adverse event, with no statistically significant difference between the groups. Both adverse events were temporary and resolved with rest and oral acetaminophen.

3: [14]	Mautner K et al. (2023)	USA	A phase 2/3, four-arm parallel, multicenter, single-blind, randomized, controlled clinical trial.	480 patients with a dx of knee OA (Kellgren–Lawrence II–IV) (4 × 120). Average age of patients in the overall study was 58.3 years. There were 214 (45.1%) males and 261 (54.9%) females who received injections.	Arm 1: autologous bone marrow aspirate concentrate (*n* = 120), CSI (*n* = 40); arm 2: umbilical cord tissue-derived mesenchymal stromal cells (*n* = 120), CSI (*n* = 40); arm 3: stromal vascular fraction (*n* = 120), CSI (*n* = 40) (97–93–103–97 completed).	120 patients were candidates for corticosteroid injection (97 completed).	Corticosteroid: A 10 mL syringe containing 1 mL of depomedrol (40 mg/dL) and 6 mL of normal saline. The BMC production procedure results in 8 mL of product with 1 mL removed and 7 mL prepared for final injection. The remaining 5 mL of the SVF were prepared for injection into the subjects. After thawing, 2–2.5 mL of solution remained and is aspirated from the cryobag, suspended in saline to produce 7 mL. which was then prepared for injection into the subject (UCT).	Of note, the BMAC and SVF were fresh autologous products, while the UCT were cryopreserved, purified MSCs manufactured from donated allogeneic umbilical cord tissue in a cGMP (current good manufacturing practice) facility.	Visual analog scale (VAS) pain score and knee injury and osteoarthritis outcome score (KOOS) pain score at 12 months versus baseline. In addition to our primary outcome measures, we also analyzed EQ-5D and PROMIS-29 between cohorts and CSI.	At 1-year postinjection, none of the three orthobiologic injections was superior to another, or to the CSI control none of the four groups showed a significant change in magnetic resonance imaging osteoarthritis score compared to baseline. For EQ-5D, the treatment by time interaction was not significant (*p*=0.26), suggesting EQ-5D in the four treatment groups changed in similar ways (similar temporal patterns for the four treatment groups). Similarly, for PROMIS-29, we assessed all domains by a treatment by time interaction, and there was no significance for the following subdomains: PROMIS-29 anxiety (*p*=0.78), depression (*p*=0.06), fatigue (*p*=0.56), pain (*p*=0.39), physical function (*p*=0.048), sleep (*p*=0.91), and social rules and activities (*p*=0.82).	There were no procedure-related serious adverse events (AEs) reported, which includes any allergic reactions or symptomatic infections seen in any treated patient. However, there were multiple related AEs reported that have been subdivided. The following related AEs demonstrated significance between cohorts: joint swelling (CSI 7.4% vs. UCT 24.1%, *p*=0.01), postprocedural contusion (SVF 38.6% vs. BMAC 12.2% versus UCT/CSI 0%, *p* < 0.0001), postprocedural hematoma (BMAC 2.9% vs. SVF 12.4%, *p*=0.02).

4: [15]	Wang Y et al. (2016)	China	Randomized controlled trial.	36 patients with moderate or severe degenerative knee OA randomized into two groups with a ratio of 1:1.	Intra-articular injection of 2.5–3.0 mL human umbilical cord MSCs suspension containing (2–3) × 10^7^ cells was performed once a month for 2 times as a course of treatment in the cell treatment group.	Sodium hyaluronate by intra-articular injection was used once a week for 5 times as a course of treatment in the control group.	2.5–3.0 mL human umbilical cord MSCs suspension containing (2–3) × 10^7^ cells.	Human umbilical cord mesenchymal stem cells.	The clinical efficacy was evaluated by SF-36 scale score, Lysholm score, and WOMAC score.	After injection, the incidences of pain and swelling in the cell treatment group were significantly higher than those in the control group (*χ*2 = 16.200, *p* < 0.001; *χ*2 = 11.688, *p* < 0.001), but no significant difference was found in the incidence of effusion (*χ*2 = 2.118, *p*=0.146). In the cell treatment group, Lysholm score at 1–6 months after treatment, WOMAC score and SF-36 scale score at 2–6 months after treatment were significantly better when compared with scores before treatment (*p* < 0.05), and no recurrence of knee pain was observed during follow-up. In the control group, there was no significant difference in Lysholm score and SF-36 scale score between pre and post-treatment (*p* > 0.05); there were significant differences in WOMAC score between pretreatment and at 1, 2, 3 months after treatment (*p* < 0.05); at 3 months after treatment, 11 patients had joint pain symptoms again. No significant difference was found in the knee joint function score and SF-36 scale score at 1 and 2 months after treatment between 2 groups (*p* > 0.05), but the scores of the cell treatment group were significantly better than those of the control group at 3 and 6 months (*p* < 0.05).	In the cell therapy group, 16 patients (88.89%) experienced pain at the injection site, and 12 patients (66.67%) had mild swelling, with pain lasting 3 to 12 h and resolving within 24 h. Local swelling typically subsided within 1 to 3 days, and most patients found these symptoms tolerable; however, 2 patients (11.11%) had intolerable pain and swelling 12 h postinjection, which was alleviated after aspirating about 30 mL of joint fluid 3. In the control group, 4 patients (22.22%) reported pain at the injection site within 30 min, and 2 patients (11.11%) had mild swelling, but these symptoms resolved within 24 h, with no joint effusion reported.

5: [16]	Ao Y et al. (2023)	China	Prospective a phase I, single-arm study.	14 patents (4 males and 10 females) with 58.29 ± 8.99 years old.	Each patient received an intra-articular injection of UC-MSCs once a week for 4 times.	No control group.	Cells were suspended in a volume of 3 mL saline with 1.5 × 10^7^ cells and loaded into a 5 mL sterile syringe for subsequent injection.	Mesenchymal derived from the umbilical cord.	Outcomes assessed by WOMAC, VAS, SF-12, and MRI results according to the magnetic resonance observation of cartilage repair tissue (MOCART) score at baseline and 3 months after the last injection. Adverse events (AEs) were documented after each injection.	Vas (6.0 [4.5–8.3] to 3.5[2.0–5.0]) and WMAC (26.0 [21.0–37.0] to 8.5 [7. 0–12.75]) scores reduced and SF-12 (39.0 [35.8–42.3] to 46.0 [44.0–48.3]) and MOCART (42.0 [34.0–48.0] to 58.0 [49.0–68.3]) score increased after injection.	The incidence of AEs was 35.7% and resolved spontaneously, and the severity of AEs was all non-serious. Most of these AEs were associated with intra-articular injection, including joint pain, swelling, numbness, and stiffness. Some other symptoms emerged during the first 3 days after injection, but all of them were transient and did not affect the patient's normal activities.

6: [17]	Soltanai KS et al. (2018)	Iran	Double-blind, placebo-controlled clinical trial.	20 patients.	Received intra-articular injection of allogeneic placenta-derived MSCs (10 ml, 0.5–0.6 × 10^8^).	10 mL of normal saline.	0.5–0.6 × 10^8^ cells of placenta-derived MSCs.	MSCs derived from placenta.	Outcomes assessed by knee flexion range of motion (ROM), VAS, and KOOS, before injection and at 2, 8, and 24 weeks after injection. Knee magnetic resonance arthrography (MRA) was used to evaluate the intra-articular structures before and 24 weeks after treatment.	ROM: significant differences were reported between the two groups at 8 and 24-week follow-ups (*p* < 0.05). In the MSC group, improvement in knee joint ROM was significant between 2 and 24 weeks and all interval pairs except for before and 2 weeks after treatment, in the control group the improvement was not significant in all interval pairs. VAS: the group–time interaction effect was not significant (*p*=0.401). KOOS: significant improvements were seen in quality of life, activity of daily living, sport/recreational activity, and decreased OA symptoms in the MSC, injected group until 8 weeks (*p* < 0.05). MRA: chondral thickness was improved in about 10% of the total knee joint area in the intervention group in 24 weeks (effect size: 0.3).	Four patients in the MSC group had increased local pain and mild effusion. Their symptoms were mild and self-limited within 48–72 h. In the 24-week clinical and radiological follow-up, there was no ectopic mass formation or any other clinical adverse effects.

7: [18]	Matas J et al. 2024	Chile	A dose–response clinical trial.	Forty participants with knee osteoarthritis aged between 30 and 75 years from both gender were finally recruited.	Intervention 1: low-dose group (2 × 10^6^ cells) (*n* = 16), intervention 2: medium-dose group (20 × 10^6^) (*n* = 16), and intervention 3: high-dose group (80 × 10^6^) (*n* = 8) received umbilical cord-derived MSCs.	No control.	Low-dose : 2 × 10^6^ cells, medium-dose: 20 × 10^6^, and high dose: 80 × 10^6^.	Umbilical cord-derived M	The primary endpoint was safety, according to the frequency of treatment-related adverse events in each group. The secondary endpoint was efficacy included pain (VAS), disability (WOMAC), and whole-organ magnetic resonance imaging score (WORMS).	According to WOMAC total outcomes, patients treated with all doses reported significant improvements in pain and function compared with baseline after 3 and 6 months. However, the improvements were higher in patients treated with both medium and low dose as compared to high dose. WORMS score did not show any significant change in cartilage or any other main descriptor.	All the doses were safe, and no serious adverse events were reported. Nonetheless, 100% of the patients injected with the high-dose experienced injection-related swelling and pain in the knee joint. Of all AEs registered due to injection, the most common was pain. Notably, patients who received the lower MSC dose have less and briefer pain.

8: [19]	Samara O et al. 2022	Jordan	A prospective, open-label, phase I/II study.	Sixteen adult patients of both genders with moderate and severe knee osteoarthritis aged 42–73 years.	Sixteen patients with advanced Kellgren stage were treated using two doses of expanded WJMSCs given 1 month apart.	No control.	The first injection, the dose ranged from 34 × 10^6^ to 50 × 10^6^ per knee, with a mean of 41.79 × 10^6^, whereas the second dose ranged from 33 × 10^6^ to 55 × 10^6^ with a mean of 44.4 × 10^6^ per knee.	Umbilical cord-derived MSCs (Wharton jelly).	The primary outcome of the study was safety with follow-up of 48 months. The secondary outcome was to evaluate the efficacy by using a normalized KOOS at 6, 12, and 48 months, and MRI assessment at 12 months.	Functional and pain improvement were observed at 12 and 48 months (*p* < 0.0001), with statistically significant improvement on MRI scans at 12 months in cartilage loss, osteophytes, bone marrow lesions, effusion and synovitis (*p* < 0.01), and highly significant improvement in subchondral sclerosis (*p* < 0.0001).	One patient developed moderate effusion and one superficial phlebitis.

**Table 3:** Risk of bias in quasi-experimental studies based on ROBINS-I.

Risk of bias	Ao (2023)	Matas (2024)	Samara (2022)
Bias due to confounding	Low	Low	Low
Bias in selection of participants into the study	Moderate	Moderate	Moderate
Bias in classification of interventions	Low	Low	Low
Bias due to deviations from intended interventions	Moderate	Moderate	Moderate
Bias due to missing data	Low	Low	Low
Bias in measurement of outcomes	Moderate	Low	Low
Bias in selection of the reported result	Low	Low	Low
Overall	Moderate	Moderate	Moderate

## Data Availability

The data are available upon request from the authors.
